# Designing rotationally invariant neural networks from PDEs and variational methods

**DOI:** 10.1007/s40687-022-00339-x

**Published:** 2022-08-04

**Authors:** Tobias Alt, Karl Schrader, Joachim Weickert, Pascal Peter, Matthias Augustin

**Affiliations:** Mathematical Image Analysis Group, Faculty of Mathematics and Computer Science, Campus E1.7, Saarland University, 66041 Saarbrücken, Germany

**Keywords:** Partial differential equations, Variational methods, Neural networks, Rotation invariance, Coupling

## Abstract

Partial differential equation models and their associated variational energy formulations are often rotationally invariant by design. This ensures that a rotation of the input results in a corresponding rotation of the output, which is desirable in applications such as image analysis. Convolutional neural networks (CNNs) do not share this property, and existing remedies are often complex. The goal of our paper is to investigate how diffusion and variational models achieve rotation invariance and transfer these ideas to neural networks. As a core novelty, we propose activation functions which couple network channels by combining information from several oriented filters. This guarantees rotation invariance within the basic building blocks of the networks while still allowing for directional filtering. The resulting neural architectures are inherently rotationally invariant. With only a few small filters, they can achieve the same invariance as existing techniques which require a fine-grained sampling of orientations. Our findings help to translate diffusion and variational models into mathematically well-founded network architectures and provide novel concepts for model-based CNN design.

## Introduction

Partial differential equations (PDEs) and variational methods are core parts of various successful model-based image processing approaches; see, e.g., [6, 11, 75] and the references therein. Such models often achieve invariance under transformations such as translations and rotations by design. These invariances reflect the physical motivation of the models: Transforming the input should lead to an equally transformed output.

Convolutional neural networks (CNNs) and deep learning [31, 42, 43, 66] have revolutionised the field of image processing in recent years. The flexibility of CNN models allows to apply them to various tasks in a plug-and-play fashion with remarkable performance. Due to their convolution structure, CNNs are shift invariant by design. However, they lack inherent rotation invariance. Proposed adaptations often inflate the network structure and rely on complex filter design with large stencils; see, e.g., [80].

In the present paper, we tackle these problems by translating rotationally invariant PDEs and their corresponding variational formulations into neural networks. This alternative view on rotation invariance within neural architectures yields novel design concepts which have not yet been explored in CNNs.

Since in the literature, multiple notions of rotation invariance exist, we define our terminology in the following. We call an operation rotationally invariant, if rotating its input yields an equally rotated output. Thus, rotation and operation are interchangeable. This notion follows the classical definition of rotation invariance for differential operators. Note that some recent CNN literature refers to this concept as equivariance.

### Our contributions

We translate PDE and variational models into their corresponding neural architectures and identify how they achieve rotation invariance. We start with simple two-dimensional diffusion models for greyscale images. Extending the connection [2, 63, 86] between explicit schemes for these models and residual networks [36] (ResNets) leads to neural activation functions which couple network channels. Their result is based on a rotationally invariant measure involving specific channels representing differential operators.

By exploring multi-channel and multiscale diffusion models, we generalise the concept of coupling to ResNeXt [84] architectures as an extension of the ResNet. Activations which couple all network channels preserve rotation invariance, but allow to design anisotropic models with a directional filtering.

We derive three central design principles for rotationally invariant neural network design, discuss their effects on practical CNNs, and evaluate their effectiveness within an experimental evaluation. Our findings transfer inherent PDE concepts to CNNs and thus help to pave the way to more model-based and mathematically well-founded learning.

### Related work

Several works connect numerical solution strategies for PDEs to CNN architectures [2, 44, 46, 55, 87] to obtain novel architectures with better performance or provable mathematical guarantees. Others are concerned with using neural networks to solve [16, 34, 59] or learn PDEs from data [45, 62, 64]. Moreover, the approximation capabilities [17, 32, 40, 71] and stability aspects [2, 10, 33, 61, 63, 70, 86] of CNNs are often analysed from a PDE viewpoint.

The connections between neural networks and variational methods have become a topic of intensive research. The idea of learning the regulariser in a variational framework has gained considerable traction and brought the performance of variational models to a new level [23, 47, 52, 58, 60]. The closely related idea of unrolling [50, 69] the steps of a minimising algorithm for a variational energy and learning its parameters has been equally prominent and successful [1, 5, 8, 13, 35, 38, 39].

We exploit and extend connections between variational models and diffusion processes [65], and their relations to residual networks [2, 63]. In contrast with our previous works [2, 4] which focussed on the one-dimensional setting and corresponding numerical algorithms, we now concentrate on two-dimensional diffusion models that incorporate different strategies to achieve rotation invariance. This allows us to transfer concepts of rotation invariance from PDEs to CNNs, which yield hitherto unexplored CNN design strategies.

A simple option to learn a rotationally invariant model is to perform data augmentation [68], where the network is trained on randomly rotated input data. This strategy, however, only approximates rotation invariance and is heavily dependent on the data at hand.

An alternative is to design the filters themselves in a rotationally invariant way, e.g. by weight restriction [12]. However, the resulting rotation invariance is too fine-grained: The filters as the smallest network component are not oriented. Thus, the model is not able to perform a directional filtering.

Other works [24, 41] create a set of rotated input images and apply filters with weight sharing to this set. Depending on the amount of sampled orientations, this can lead to large computational overhead.

An elegant solution for inherent rotation invariance is based on symmetry groups. Gens and Domingos [28] as well as Dieleman et al. [21] propose to consider sets of feature maps which are rotated versions of each other. This comes at a high memory cost as four times as many feature maps need to be processed. Marcos et al. [49] propose to rotate the filters instead of the features, with an additional pooling of orientations. However, the pooling reduces the directional information too quickly. A crucial downside of all these approaches is that they only use four orientations. This only yields a coarse approximation of rotation invariance.

This idea has been generalised to arbitrary symmetry groups by Cohen and Welling [15] through the use of group convolution layers. Group convolutions lift the standard convolution to other symmetry groups which can also include rotations, thus leading to rotation invariance by design. However, also there, only four rotations are considered. This is remedied by Weiler et al. [78, 80] who make use of steerable filters [27] to design a larger set of oriented filters. Duits et al. [22] go one step further by formulating all layers as solvers to parametrised PDEs. Similar ideas have been implemented with wavelets [67] and circular harmonics [83], and the group invariance concept has also been extended to higher dimensional data [14, 57, 79]. However, processing multiple orientations in dedicated network channels inflates the network architecture, and discretising the large set of oriented filters requires the use of large stencils.

We provide an alternative by means of a more sophisticated activation function design. By coupling specific network channels, we can achieve inherent rotation invariance without using large stencils or group theory, while still allowing for models to perform directional filtering. In a similar manner, Mrázek and Weickert proposed to design rotationally invariant wavelet shrinkage [51] by using a coupling wavelet shrinkage function. However, to the best of our knowledge coupling activation functions have not been considered in CNNs so far.

### Organisation of the paper

We motivate our view on rotationally invariant design with a tutorial example in Sect. 2. Afterward, we review variational models and residual networks as the two other basic concepts in Sect. 3. In Sect. 4, we connect various diffusion models and their associated energies to their neural counterparts and identify central concepts for rotation invariance. We summarise our findings and discuss their practical implementation in Sect. 5 and conduct experiments on rotation invariance in Sect. 6. We finish the paper with our conclusions in Sect. 7.

## Two views on rotational invariance

To motivate our viewpoint on rotationally invariant model design, we review a nonlinear diffusion filter of Weickert [73] for image denoising and enhancement. It achieves anisotropy by integrating one-dimensional diffusion processes over all directions. This integration model creates a family of greyscale images $u(\varvec{x}, t): \varOmega \times [0, \infty ) \rightarrow \mathbb {R}$ on an image domain $\varOmega \subset \mathbb {R}^2$ according to the integrodifferential equation

1 $$\begin{aligned} \partial _t u = \frac{2}{\pi } \int _{0}^{\pi } \partial _{e_\theta } \left( g\left( \left| \partial _{e_\theta } u_\sigma \right| ^2\right) \partial _{e_\theta } u\right) \, \mathrm{d}\theta , \end{aligned}$$

where $\partial _{e_\theta }$ is a directional derivative along the orientation of an angle $\theta$. The evolution is initialised as $u(\cdot ,0) = f$ with the original image *f*, and reflecting boundary conditions are imposed. The model integrates one-dimensional nonlinear diffusion processes with different orientations $\theta$. All of them share a nonlinear decreasing diffusivity function *g* which steers the diffusion in dependence of the local directional image structure $\left| \partial _{e_\theta } u_\sigma \right| ^2$. Here, $u_\sigma$ is a smoothed version of *u* which has been convolved with a Gaussian of standard deviation $\sigma$.

As this model diffuses more along low contrast directions than along high contrast ones, it is anisotropic. It is still rotationally invariant, since it combines all orientations of the one-dimensional processes with equal importance. However, this concept comes at the cost of an elaborate discretisation. First, one requires a large amount of discrete rotation angles for a reasonable approximation of the integration. Discretising the directional derivatives in all these directions with a sufficient order of consistency requires the use of large filter stencils; cf. also [9]. The design of rotationally invariant networks such as [80] faces similar difficulties. Processing the input by applying several rotated versions of an oriented filter requires large stencils and many orientations.

A much simpler option arises when considering the closely related edge-enhancing diffusion (EED) model [74]

2 $$\begin{aligned} \partial _t u = \varvec{\nabla }^\top \left( \varvec{D}\left( \varvec{\nabla }u_\sigma \right) \varvec{\nabla }u\right) , \end{aligned}$$

where $\varvec{\nabla }= \left( \partial _x, \partial _y\right) ^\top$ denotes the gradient operator and $\varvec{\nabla }^\top$ is the divergence. Instead of an integration, the right-hand side is given in divergence from. Thus, the process is now steered by a diffusion tensor $\varvec{D} \left( \varvec{\nabla }u_\sigma \right)$. It is a $2\times 2$ positive semi-definite matrix which is designed to adapt the diffusion process to local directional information by smoothing along, but not across dominant image structures. This is achieved by constructing $\varvec{D}$ from its normalised eigenvectors $\varvec{v}_1 \parallel \varvec{\nabla }u_\sigma$ and $\varvec{v}_2 \bot \varvec{\nabla }u_\sigma$ which point across and along local structures. The corresponding eigenvalues $\lambda _1 = g\left( \left| \varvec{\nabla }u_\sigma \right| ^2\right)$ and $\lambda _2=1$ inhibit diffusion across dominant structures and allow smoothing along them. Thus, the diffusion tensor can be written as

3 $$\begin{aligned} \varvec{D} \left( \varvec{\nabla }u_\sigma \right) = g\left( \left| \varvec{\nabla }u_\sigma \right| ^2\right) \varvec{v}_1 \varvec{v}_1^\top + 1 \, \varvec{v}_2 \varvec{v}_2^\top . \end{aligned}$$

Discretising the EED model (2) is much more convenient. For example, a discretisation of the divergence term with good rotation invariance can be performed on a $3\times 3$ stencil, which is the minimal size for a consistent discretisation of a second order model [77].

This illustrates a central insight: *One can replace a complex discretisation by a sophisticated design of the nonlinearity*. This motivates us to investigate how rotationally invariant design principles of diffusion models translate into novel activation function designs.

## Review: variational methods and residual networks

We now briefly review variational methods and residual networks as the other two central concepts in our work.

### Variational regularisation

Variational regularisation [72, 82] obtains a function $u(\varvec{x})$ on a domain $\varOmega$ as the minimiser of an energy functional. A general form of such a functional reads

4 $$\begin{aligned} E(u) = \int _\varOmega \left( D\left( u,f\right) + \alpha R\left( u\right) \right) \, \mathrm{d}\varvec{x}. \end{aligned}$$

Therein, a data term *D*(*u*, *f*) drives the solution *u* to be close to an input image *f*, and a regularisation term *R*(*u*) enforces smoothness conditions on the solution. The balance between the terms is controlled by a positive smoothness parameter $\alpha$.

We restrict ourselves to energy functionals with only a regularisation term and interpret the gradient descent to the energy as a parabolic diffusion PDE. This connection serves as one foundation for our findings. The variational framework is the simplest setting for analysing invariance properties, as these are automatically transferred to the corresponding diffusion process.

### Residual networks

Residual networks (ResNets) [36] belong to the most popular neural network architectures to date. Their specific structure facilitates the training of very deep networks and shares a close connection to PDE models.

ResNets consist of chained residual blocks. A single residual block computes a discrete output $\varvec{u}$ from an input $\varvec{f}$ by means of

5 $$\begin{aligned} \varvec{u} = \varphi _2\left( \varvec{f} + \varvec{W}_2\, \varphi _1\left( \varvec{W}_1 \varvec{f} + \varvec{b}_1\right) + \varvec{b}_2\right) . \end{aligned}$$

First, one applies an inner convolution to $\varvec{f}$, which is modelled by a convolution matrix $\varvec{W}_1$. In addition, one adds a bias vector $\varvec{b}_1$. The result of this inner convolution is fed into an inner *activation* function $\varphi _1$. Often, these activations are fixed to simple functions such as the rectified linear unit (ReLU) [53] which is a truncated linear function:

6 $$\begin{aligned} \text {ReLU}(s) = \text {max}(0,s). \end{aligned}$$

The activated result is convolved with an outer convolution $\varvec{W}_2$ with a bias vector $\varvec{b}_2$. Crucially, the result of this convolution is added back to the original input signal $\varvec{f}$. This *skip connection* is the key to the success of ResNets, as it avoids the vanishing gradient phenomenon found in deep feed-forward networks [7, 36]. Lastly, one applies an outer activation function $\varphi _2$ to obtain the output $\varvec{u}$ of the residual block.

In contrast with diffusion processes and variational methods, these networks are not committed to a specific input dimensionality. In standard networks, the input is quickly deconstructed into multiple channels, each one concerned with different, specific image features. Each channel is activated independently, and information is exchanged through trainable convolutions. While this makes networks flexible, it does not take into account concepts such as rotation invariance. By translating rotationally invariant diffusion models into ResNets and extensions thereof, we will see that shifting the focus from the convolutions towards activations can serve as an alternative way to guarantee built-in rotation invariance within a network.

## From diffusion PDEs and variational models to rotationally invariant networks

With the concepts from Sects. 2 and 3 , we are now in a position to derive diffusion-inspired principles of rotationally invariant network design.

### Isotropic diffusion on greyscale images

We first consider the simplest setting of isotropic diffusion models for images with a single channel. By reviewing three popular models, we identify the common concepts for rotation invariance, and find a unifying neural network interpretation.

We start with the second order diffusion model of Perona and Malik [56], which is given by the PDE

7 $$\begin{aligned} \partial _t u = \varvec{\nabla }^\top \left( g\left( \left| \varvec{\nabla }u\right| ^2\right) \varvec{\nabla }u\right) , \end{aligned}$$

with reflecting boundary conditions. This model creates a family of gradually simplified images $u(\varvec{x}, t)$ according to the diffusivity $g(s^2)$. It attenuates the diffusion at locations where the gradient magnitude of the evolving image is large. In contrast with the model of Weickert (1), the Perona–Malik model is isotropic, i.e. it does not have a preferred direction.

The variational counterpart of this model helps us to identify the cause of its rotation invariance. An energy for the Perona–Malik model can be written in the following way which allows different generalisations:

8 $$\begin{aligned} E(u) = \int _{\varOmega } \varPsi \left( \text {tr}\left( \varvec{\nabla }u \varvec{\nabla }u^\top \right) \right) \, \mathrm{d}\varvec{x}, \end{aligned}$$

with an increasing regulariser function $\varPsi$ which can be connected to the diffusivity *g* by $g=\varPsi ^\prime$ [65]. Comparing the functional 8 to the one in (4), we have now specified the form of the regulariser to be $R(u) =\varPsi \left( \text {tr}\left( \varvec{\nabla }u \varvec{\nabla }u^\top \right) \right)$.

The argument of the regulariser is the trace of the so-called structure tensor [26], here without Gaussian regularisation, which reads

9 $$\begin{aligned} \varvec{\nabla }u \varvec{\nabla }u^\top = \begin{pmatrix} u_{x}^2 &{} u_{x}u_{y}\\ u_{x}u_{y} &{} u_{y}^2 \end{pmatrix}. \end{aligned}$$

This structure tensor is a $2 \times 2$ matrix with eigenvectors $\varvec{v}_1 \parallel \varvec{\nabla }u$ and $\varvec{v}_2 \bot \varvec{\nabla }u$ parallel and orthogonal to the image gradient. The corresponding eigenvalues are given by $\nu _1 = \left| \varvec{\nabla }u\right| ^2$ and $\nu _2 = 0$, respectively. Thus, the eigenvectors span a local coordinate system where the axes point across and along dominant structures of the image, and the larger eigenvalue describes the magnitude of image structures.

The use of the structure tensor is the key to rotation invariance. A rotation of the image induces a corresponding rotation of the structure tensor and the structural information that it encodes: Its eigenvectors rotate along, and its eigenvalues remain unchanged. Consequently, the trace as the sum of the eigenvalues is rotationally invariant.

In the following, we explore other ways to design the energy functional based on rotationally invariant quantities and investigate how the resulting diffusion model changes.

The fourth-order model of You and Kaveh [85] relies on the Hessian matrix. The corresponding energy functional reads

10 $$\begin{aligned} E(u) = \int _{\varOmega } \varPsi \left( \left( \text {tr}\left( \varvec{H}(u)\right) \right) ^2\right) \, \mathrm{d}\varvec{x}. \end{aligned}$$

Here, the regulariser takes the squared trace of the Hessian matrix $\varvec{H}(u)$ as an argument. Since the trace of the Hessian is equivalent to the Laplacian $\varDelta u$, the gradient flow of (10) can be written as

11 $$\begin{aligned} \partial _t u = - \varDelta \left( g\left( \left( \varDelta u\right) ^2\right) \varDelta u\right) . \end{aligned}$$

This is a fourth-order counterpart to the Perona–Malik model. Instead of the gradient operator, one considers the Laplacian $\varDelta$. This change was motivated as one remedy to the staircasing effect of the Perona–Malik model [85].

The rotationally invariant matrix at hand is the Hessian $\varvec{H}(u)$. In a similar manner as the structure tensor, the Hessian describes local structure and thus follows a rotation of this structure. Also in this case, the trace operation reduces the Hessian to a scalar that does not change under rotations.

To avoid speckle artefacts of the model of You and Kaveh, Lysaker et al. [48] propose to combine all entries of the Hessian in the regulariser. They choose the Frobenius norm of the Hessian $||\varvec{H}(u)||^2_F$ together with a total variation regulariser. For more general regularisers, this model reads [20]

12 $$\begin{aligned} E(u) = \int _{\varOmega } \varPsi \left( ||\varvec{H}(u)||^2_F\right) \, \mathrm{d}\varvec{x}, \end{aligned}$$

which yields a diffusion process of the form

13 $$\begin{aligned} \partial _t u = - \mathcal {D}^\top \left( g\left( ||\varvec{H}(u)||^2_F\right) \mathcal {D} u\right) , \end{aligned}$$

where the differential operator ${\mathcal {D}}$ induced by the Frobenius norm reads

14 $$\begin{aligned} {\mathcal {D}} = \left( \partial _{xx}, \partial _{xy}, \partial _{yx}, \partial _{yy}\right) ^\top . \end{aligned}$$

This shows another option how one can use the rotationally invariant information of the Hessian matrix. While the choice of a Frobenius norm instead of the trace operator changes the associated differential operators in the diffusion model, it does not destroy the rotation invariance property: The squared Frobenius norm is the sum of the squared eigenvalues of the Hessian, which in turn are rotationally invariant.

### Coupled activations for operator channels

In the following, we extend the connections between residual networks and explicit schemes from [2, 63, 86] in order to transfer rotation invariance concepts to neural networks. To this end, we consider the generalised diffusion PDE

15 $$\begin{aligned} \partial _t u = - \mathcal {D}^* \left( g\left( |{\mathcal {D}} u|^2\right) {\mathcal {D}} u\right) . \end{aligned}$$

Here, we use a generalised differential operator ${\mathcal {D}}$ and its adjoint ${\mathcal {D}}^*$. This PDE subsumes the diffusion models (7), (11), and (13). Since the diffusivities take a scalar argument, we can express the diffusivity as $g(|{\mathcal {D}} u|^2)$. The differential operator $\mathcal D$ is induced by the associated energy functional.

To connect the generalised model (15) to a ResNet architecture, we first rewrite (15) by means of the vector-valued flux function $\varvec{\varPhi }(\varvec{s}) = g(|\varvec{s}|^2)\, \varvec{s}$ as

16 $$\begin{aligned} \partial _t u = - \mathcal {D}^* \left( \varvec{\varPhi }\left( \mathcal {D} u\right) \right) . \end{aligned}$$

Let us now consider an explicit discretisation for this diffusion PDE. The temporal derivative is discretised by a forward difference with time step size $\tau$, and the spatial derivative operator $\mathcal {D}$ is discretised by a convolution matrix $\varvec{K}$. Consequently, the adjoint $\mathcal {D}^*$ is discretised by $\varvec{K}^\top$. Depending on the number of components of $\mathcal {D}$, the matrix $\varvec{K}$ implements a set of convolutions. This yields an explicit scheme for (16)

17 $$\begin{aligned} \varvec{u}^{k+1} = \varvec{u}^k - \tau \varvec{K}^\top \varvec{\varPhi }\left( \varvec{K} \varvec{u}^k\right) . \end{aligned}$$

where a superscript *k* denotes the discrete time level. One can connect this explicit step (17) to a residual block (5) by identifying

18 $$\begin{aligned} \varvec{W}_1 = \varvec{K}, \quad \varphi _1 = \tau \varvec{\varPhi }, \quad \varvec{W}_2 = -\varvec{K}^\top , \quad \varphi _2 = \text {Id}, \end{aligned}$$

and setting the bias vectors to $\varvec{0}$ [2, 63, 86].

In contrast with the one-dimensional considerations in [2], the connection between flux function and activation in the two-dimensional setting yields additional, novel design concepts for activation functions. This yields the first design principle for neural networks.

#### Design Principle 1

 Activation functions which couple network channels can be used to design rotationally invariant networks. At each position of the image, the channels of the inner convolution result are combined within a rotationally invariant quantity which determines the nonlinear response.

The coupling effect of the diffusivity and the regulariser directly transfers to the activation function. This is apparent when the differential operator ${\mathcal {D}}$ contains multiple components. For example, consider an operator ${\mathcal {D}} = \left( {\mathcal {D}}_1, {\mathcal {D}}_2\right) ^\top$ with two components and its discrete variant $\varvec{K} = \left( \varvec{K}_1, \varvec{K}_2\right) ^\top$.

The application of the operator $\varvec{K}$ transforms the single-channel signal $\varvec{u}^k$ into a signal with two channels. Then the activation function couples the information from both channels within the diffusivity *g*. For each pixel position *i*, *j*, we have

19 $$\begin{aligned} \varvec{\varPhi }\begin{pmatrix} \left( \varvec{K}_1 \varvec{u}^k\right) _{i,j} \\ \left( \varvec{K}_2 \varvec{u}^k\right) _{i,j} \end{pmatrix} = g\left( \left| \varvec{K}_1 \varvec{u}^k\right| _{i,j}^2 + \left| \varvec{K}_2 \varvec{u}^k\right| _{i,j}^2 \right) \begin{pmatrix} \left( \varvec{K}_1 \varvec{u}^k\right) _{i,j} \\ \left( \varvec{K}_2 \varvec{u}^k\right) _{i,j} \end{pmatrix}. \end{aligned}$$

Afterwards, the application of $\varvec{K}^\top$ reduces the resulting two-channel signal to a single channel again.

In the general case, the underlying differential operator ${\mathcal {D}}$ determines how many channels are coupled. The choice ${\mathcal {D}} = \left( \partial _{xx}, \partial _{xy}, \partial _{yx}, \partial _{yy}\right) ^\top$ of Lysaker et al. [48] induces a coupling of four channels containing second order derivatives. This shows that a central condition for rotation invariance is that the convolution $\varvec{K}$ implements a rotationally invariant differential operator. We discuss the effects of this condition on the practical filter design in Sect. 5.

**Fig. 1:**
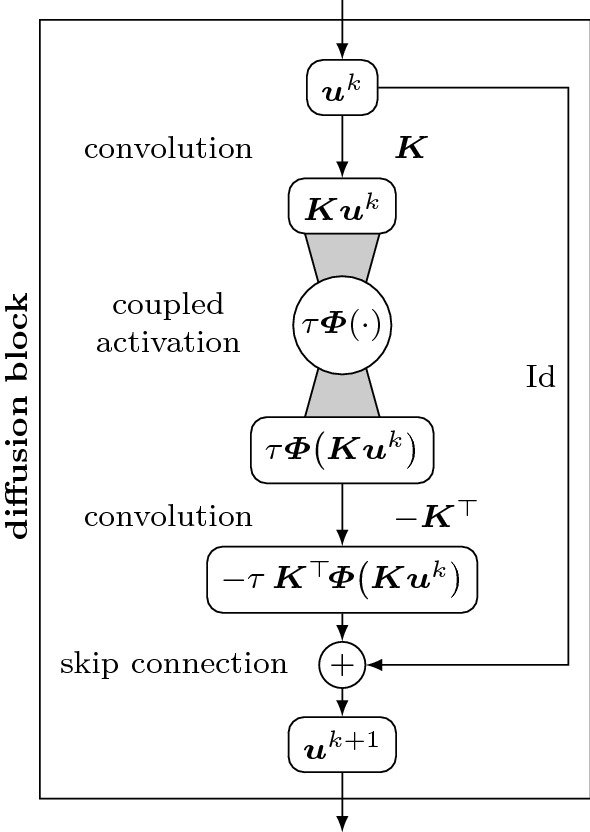
Diffusion block for an explicit diffusion step (17) with activation function $\tau \varvec{\varPhi }$, time step size $\tau$, and convolution filters $\varvec{K}$. The activation function couples the channels of the operator $\varvec{K}$

We call a block of the form (17) a *diffusion block*. It is visualised in Fig. 1 in graph form. Nodes contain the state of the signal, while edges describe the operations to move from one state to another. We denote the channel coupling by a shaded connection to the activation function.

The coupling effect is natural in the diffusion case. However, to the best of our knowledge, this concept has not been proposed for CNNs in the context of rotation invariance.

### Diffusion on multi-channel images

So far, the presented models have been isotropic. They only consider the magnitude of local image structures, but not their direction. However, we will see that anisotropic models inspire another form of activation function which combines directional filtering with rotation invariance.

To this end, we move to diffusion on multi-channel images. While there are anisotropic models for single-channel images [75], they require a presmoothing as shown in the EED model (2). However, such models do not have a conventional energy formulation [81]. The multi-channel setting allows one to design anisotropic models that do not require a presmoothing and arise from a variational energy.

In the following, we consider multi-channel images $\varvec{u} = \left( u_1, u_2, \dots , u_M\right) ^\top$ with *M* channels. To distinguish them from the previously considered channels of the differential operator, we refer to image channels and operator channels in the following.

A naive extension of the Perona–Malik model (7) to multi-channel images would treat each image channel separately. Consequently, the energy would consider a regularisation of the trace of the structure tensor for each individual channel. This in turn does not respect the fact that structural information is correlated in the channels.

To incorporate this correlation, Gerig et al. [29] proposed to sum up structural information from all channels. An energy functional for this model reads20Here, we again use the trace formulation. It shows that this model makes use of a colour structure tensor, which goes back to Di Zenzo [18]. It is the sum of the structure tensors of the individual channels. In contrast with the single-channel structure tensor without Gaussian regularisation, no closed form solution for its eigenvalues and eigenvectors are available. Still, the sum of structure tensors stays rotationally invariant.

The corresponding diffusion process is described by the coupled PDE set

21 $$\begin{aligned} \partial _t u_m = \varvec{\nabla }^\top \left( g\left( \sum _{n=1}^{M}\left| \varvec{\nabla }u_n\right| ^2\right) \varvec{\nabla }u_m\right) \qquad \left( m=1,\dots ,M\right) , \end{aligned}$$

with reflecting boundary conditions. As trace and summation are interchangeable, the argument of the regulariser corresponds to a sum of channel-wise gradient magnitudes. Thus, the diffusivity considers information from all channels. It allows to steer the diffusion process in all channels depending on a joint structure measure.

Interestingly, a simple change in the energy model (20) incorporates directional information [76] such that the model becomes anisotropic. Switching the trace operator and the regulariser yields the energy22Now the regulariser acts on the colour structure tensor in the sense of a power series. Thus, the regulariser modifies the eigenvalues $\nu _1, \nu _2$ to $\varPsi \left( \nu _1\right) , \varPsi \left( \nu _2\right)$ and leaves the eigenvectors unchanged. For the $2\times 2$ colour structure tensor we have

23 $$\begin{aligned} \varPsi \left( \sum _{m=1}^M\varvec{\nabla }u_m \varvec{\nabla }u_m^\top \right) = \varPsi \left( \nu _1\right) \varvec{v}_1 \varvec{v}_1^\top + \varPsi \left( \nu _2\right) \varvec{v}_2 \varvec{v}_2^\top . \end{aligned}$$

The eigenvalues are treated individually. This allows for an anisotropic model, as each eigenvalue determines the local image contrast along its corresponding eigenvector. Still, the model is rotationally invariant as the colour structure tensor rotates accordingly. Consequently, the trace of this regulariser is equivalent to the sum of the regularised eigenvalues:

24 $$\begin{aligned} \text {tr }\varPsi \left( \sum _{m=1}^M\varvec{\nabla }u_m \varvec{\nabla }u_m^\top \right) = \varPsi \left( \nu _1\right) + \varPsi \left( \nu _2\right) . \end{aligned}$$

This illustrates the crucial difference to the isotropic case, where we have

25 $$\begin{aligned} \varPsi \left( \text {tr }\sum _{m=1}^M\varvec{\nabla }u_m \varvec{\nabla }u_m^\top \right) = \varPsi \left( \nu _1 + \nu _2\right) , \end{aligned}$$

Both eigenvalues of the structure tensor are regularised jointly and the result is a scalar, which shows that no directional information can be involved. At this point, the motivation for using the structure tensor notation in the previous models becomes apparent: Switching the trace operator and the regulariser changes an isotropic model into an anisotropic one.

The gradient descent of the energy (22) is an anisotropic nonlinear diffusion model for multi-channel images [76]:

26 $$\begin{aligned} \partial _t u_m = \varvec{\nabla }^\top \left( g\left( \sum _{n=1}^M\varvec{\nabla }u_n \varvec{\nabla }u_n^\top \right) \varvec{\nabla }u_m\right) \qquad \left( m=1,\dots ,M\right) . \end{aligned}$$

The diffusivity inherits the matrix-valued argument of the regulariser. Thus, it is applied in the same way and yields a $2\times 2$ diffusion tensor. In contrast to single-channel diffusion, this creates anisotropy as its eigenvectors do not necessarily coincide with $\varvec{\nabla }u$. Thus, the multi-channel case does not require Gaussian presmoothing.

We have seen that the coupling effect within the diffusivity goes beyond the channels of the differential operator. It combines both the operator channels as well as the image channels within a joint measure. Whether the model is isotropic or anisotropic is determined by the shape of the diffusivity result: Isotropic models use scalar diffusivities, while anisotropic models require matrix-valued diffusion tensors. In the following, we generalise this concept and analyse its influence on the ResNet architecture.

### Coupled activations for image channels

A generalised formulation of the multichannel diffusion models (21) and (26) is given by

27 $$\begin{aligned} \partial _t u_m = - \mathcal {D}^* \varvec{\varPhi }\left( \varvec{u}, \mathcal {D} u_m\right) \qquad \left( m=1,\dots ,M\right) . \end{aligned}$$

As the flux function uses more information than only $\mathcal {D} u_m$, we switch to the notation $\varvec{\varPhi }\left( \varvec{u}, \mathcal {D} u_m\right)$. An explicit scheme for this model is derived in a similar way as before, yielding

28 $$\begin{aligned} \varvec{u}_m^{k+1} = \varvec{u}_m^k - \tau \varvec{K}^\top \varvec{\varPhi } \left( \varvec{u}^k, \varvec{K} \varvec{u}_m^k\right) \qquad \left( m=1,\dots ,M\right) . \end{aligned}$$

The activation function now couples more than just the operator channels, it couples all its input channels. In contrast to Design Principle 1, this coupling is more general and provides a second design principle.

#### Design Principle 2

(Fully Coupled Activations for Image Channels) Activations which couple both operator channels and image channels can be used to create anisotropic, rotationally invariant models. At each position of the image, all operator channels for all image channels are combined within a rotationally invariant quantity which determines the nonlinear response.

Different coupling effects serve different purposes: Coupling the image channels accounts for structural correlations and can be used to create anisotropy. Coupling the channels of the differential operators guarantees rotation invariance.

This design principle becomes apparent when explicitly formulating the activation function. Isotropic models use a scalar diffusivity within the flux function

29 $$\begin{aligned} \varvec{\varPhi } \left( \varvec{u}^k, \left( \varvec{K} \varvec{u}_m^k\right) _{i,j}\right) = g\left( \sum _{m=1}^M\left| \varvec{K} \varvec{u}^k_m\right| ^2_{i,j}\right) \left( \varvec{K} \varvec{u}^k_m\right) _{i,j} \qquad \left( m=1,\dots ,M\right) , \end{aligned}$$

which couples all channels of $\varvec{u}$ at the position *i*, *j*, as well as all components of the discrete operator $\varvec{K}$. Anisotropic models require a matrix-valued diffusion tensor in the flux function

30 $$\begin{aligned} \varvec{\varPhi } \left( \varvec{u}^k , \left( \varvec{K} \varvec{u}_m^k\right) _{i,j}\right) = g\left( \sum _{m=1}^M \left( \varvec{K} \varvec{u}^k_m\right) _{i,j} \left( \varvec{K} \varvec{u}^k_m\right) _{i,j}^\top \right) \left( \varvec{K} \varvec{u}^k_m\right) _{i,j} \,\, \left( m=1,\dots ,M\right) , \end{aligned}$$

**Fig. 2:**
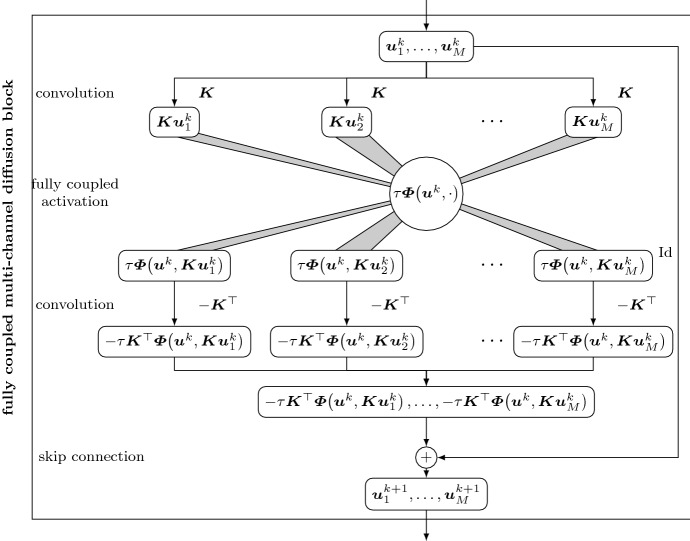
Fully coupled multi-channel diffusion block for an explicit step (28) with a fully coupled activation function $\tau \varvec{\varPhi }$, time step size $\tau$, and convolution filters $\varvec{K}$. The activation function couples all operator and image channels of its input jointly. Depending on the design of the activation, the resulting model can be isotropic or anisotropic

This concept is visualised in Fig. 2 in the form of a *fully coupled multi-channel diffusion block*. To clarify the distinction between image and operator channels, we explicitly split the image into its channels. We see that all information of the inner filter passes through a single activation function and influences all outgoing results in the same manner.

Design Principle 2 shows that coupling cannot only be used for rotationally invariant design, but also makes sense for implementing modelling aspects such as anisotropy. This is desirable as anisotropic models often exhibit higher performance through better adaptivity to data.

### Integrodifferential diffusion

The previous models work on the finest scale of the image. However, image structures live on different scales of the image. Large image structures are present on coarser scales than fine ones. Generating a structural measure which incorporates information from multiple image scales can be beneficial.

To this end, we consider integrodifferential extensions of single scale diffusion which have proven advantageous in practical applications such as denoising [3]. In analogy to the multi-channel diffusion setting, these models inspire a full coupling of scale information for a variation in residual networks.

We start with the energy functional

31 $$\begin{aligned} E\left( u\right) = \int _\varOmega \varPsi \left( \text {tr }\int _{0}^{\infty } \left( {\mathcal {D}}^{(\sigma )} u\right) \left( {\mathcal {D}}^{(\sigma )} u\right) ^\top \mathrm{d}\sigma \right) \, \mathrm{d}\varvec{x}. \end{aligned}$$

We denote the scale parameter by $\sigma$ and assume that the differential operators ${\mathcal {D}}^{(\sigma )}$ are dependent on the scale. This can be realised for example by an adaptive presmoothing of an underlying differential operator; see e.g. [3, 19].

Instead of summing structure tensors over image channels, this model integrates generalised structure tensors $\left( {\mathcal {D}}^{(\sigma )} u\right) \left( {\mathcal {D}}^{(\sigma )} u\right) ^\top$ over multiple scales. This results in a multiscale structure tensor [3] which contains a semi-local measure for image structure. If ${\mathcal {D}}^{(\sigma )}$ are rotationally invariant operators, then the multiscale structure tensor is also invariant.

The corresponding diffusion model reads

32 $$\begin{aligned} \partial _t u = - \int _{0}^{\infty }\mathcal {D}^{(\sigma )*} \left( g\left( \int _{0}^{\infty }\left| {\mathcal {D}}^{(\gamma )} u\right| ^2 \, \mathrm{d}\gamma \right) \mathcal {D}^{(\sigma )} u \right) \, \mathrm{d}\sigma , \end{aligned}$$

where $g = \varPsi ^\prime$. Due to the chain rule, one obtains two integrations over the scales: The outer integration combines diffusion processes on each scale. The inner integration, where the scale variable has been renamed to $\gamma$, accumulates multiscale information within the diffusivity argument.

This model is a variant of the integrodifferential isotropic diffusion model of Alt and Weickert [3]. Therein, however, the diffusivity uses a scale-adaptive contrast parameter. Thus, it does not arise from an energy functional.

As in the multi-channel diffusion models, switching trace and regulariser yields an anisotropic model, which is described by the energy

33 $$\begin{aligned} E\left( u\right) = \int _\varOmega \text {tr }\varPsi \left( \int _{0}^{\infty }\left( {\mathcal {D}}^{(\sigma )} u\right) \left( {\mathcal {D}}^{(\sigma )} u\right) ^\top \mathrm{d}\sigma \right) \, \mathrm{d}\varvec{x}. \end{aligned}$$

In analogy to the multi-channel model, the regulariser is applied directly to the structure tensor, which creates anisotropy. Consequently, the resulting diffusion process is a variant of the integrodifferential anisotropic diffusion [3]:

34 $$\begin{aligned} \partial _t u = - \int _{0}^{\infty }\mathcal {D}^{(\sigma )*} \left( g\left( \int _{0}^{\infty }\left( \mathcal {D}^{(\gamma )} u\right) \left( {\mathcal {D}}^{(\gamma )} u\right) ^\top \mathrm{d}\gamma \right) \mathcal {D}^{(\sigma )} u\right) \mathrm{d}\sigma . \end{aligned}$$

The anisotropic regularisation is inherited by the diffusivity and results in a flux function that implements a matrix-vector multiplication.

### Coupled activations for image scales

Both the isotropic and the anisotropic multiscale models can be summarised by the flux formulation

35 $$\begin{aligned} \partial _t u = - \int _{0}^{\infty }\mathcal {D}^{(\sigma )*} \left( \varvec{\varPhi }\left( u, \mathcal {D}^{(\sigma )} u\right) \right) \, \mathrm{d}\sigma . \end{aligned}$$

To discretise this model, we now require a discretisation of the scale integral. To this end, we select a set of *L* discrete scales $\sigma _1, \sigma _2, \dots , \sigma _L$. On each scale $\sigma _\ell$, we employ discrete differential operators $\varvec{K}_\ell$. This yields an explicit scheme for the continuous model (35) which reads

36 $$\begin{aligned} \varvec{u}^{k+1} = \varvec{u}^k - \tau \sum _{\ell =1}^{L}\omega _\ell \, \varvec{K}_\ell ^\top \varvec{\varPhi } \left( \varvec{u}^k, \varvec{K}_\ell \varvec{u}^k\right) . \end{aligned}$$

Here, $\omega _\ell$ is a step size over the scales, discretising the infinitesimal quantity $\mathrm{d}\sigma$. It is dependent on the scale to allow a non-uniform sampling of scales $\sigma _\ell$. A simple choice is $\omega _\ell = \sigma _{\ell +1} - \sigma _{\ell }$.

Interestingly, an extension of residual networks called ResNeXt [84] provides the corresponding neural architecture to this model. Therein, the authors consider a sum of transformations of the input signal together with a skip connection. We restrict ourselves to the following formulation:

37 $$\begin{aligned} \varvec{u} = \varphi _2\left( \varvec{f} + \sum _{\ell = 1}^{L} \varvec{W}_{2,\ell } \, \varphi _\ell \left( \varvec{W}_{1,\ell } \varvec{f} + \varvec{b}_{1,\ell }\right) + \varvec{b}_{2,\ell }\right) . \end{aligned}$$

This ResNeXt block modifies the input image $\varvec{f}$ within *L* independent paths and sums up the results before the skip connection. Each path may apply multiple, differently shaped convolutions. Choosing a single path with $L=1$ yields the ResNet model.

We can identify an explicit multiscale diffusion step (36) with a ResNeXt block (37) by

38 $$\begin{aligned} \varvec{W}_{1,\ell } = \varvec{K}_\ell , \quad \varphi _{1,\ell } = \tau \, \varvec{\varPhi }, \quad \varvec{W}_{2,\ell } = -\omega _\ell \varvec{K}^\top _\ell , \quad \varphi _2 = \text {Id}, \end{aligned}$$

and all bias vectors $\varvec{b}_{1,\ell }, \varvec{b}_{2,\ell }$ are set to $\varvec{0}$, for all $\ell =1,\dots ,L$.

In contrast with the previous ResNet relation (18), we apply different filters $\varvec{K}_\ell$ in each path. Their individual results are summed up before the skip connection, which approximates the scale integration. While the ResNeXt block allows for individual activation functions in each path, we use a common activation with a full coupling for all of them. This constitutes a variant of Design Principle 2, where one now couples image scales.

#### Design Principle 3

(Fully Coupled Activations for Image Scales) Activations which couple both operator channels and image scales can be used to create anisotropic, rotationally invariant multiscale models. At each position of the image, all operator channels for all image scales are combined within a rotationally invariant quantity which determines the nonlinear response.

**Fig. 3:**
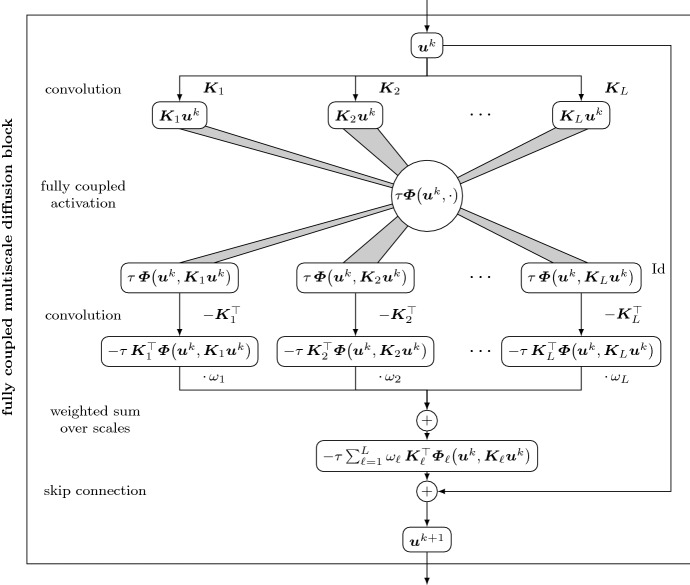
Fully coupled multiscale diffusion block for an explicit multiscale diffusion step (36) with a single activation function $\tau \omega _\ell \varvec{\varPhi }$, time step size $\tau$, and convolution filters $\varvec{K}_\ell$ on each scale. The activation function couples all inputs jointly. Depending on the design of the activation, the resulting model can be isotropic or anisotropic

Also in this case, the combined coupling serves different purposes. Coupling the operator channels yields rotation invariance, and coupling of scales allows to obtain a more global representation of the image structure. Isotropic models employ a coupling with a scalar diffusivity in the flux function

39 $$\begin{aligned} \varvec{\varPhi }\left( \varvec{u}^k, \left( \varvec{K}_\ell \varvec{u}^k\right) _{i,j}\right) = g\left( \sum _{\ell =1}^L\left| \varvec{K}_\ell \varvec{u}^k\right| _{i,j}^2 \right) \left( \varvec{K}_\ell \varvec{u}^k\right) _{i,j}, \end{aligned}$$

and a matrix-valued diffusion tensor in the flux function

40 $$\begin{aligned} \varvec{\varPhi }\left( \varvec{u}^k, \left( \varvec{K}_\ell \varvec{u}^k\right) _{i,j}\right) = g\left( \sum _{\ell =1}^L\left( \varvec{K}_\ell \varvec{u}^k\right) _{i,j} \left( \varvec{K}_\ell \varvec{u}^k\right) _{i,j}^\top \right) \left( \varvec{K}_\ell \varvec{u}^k\right) _{i,j} \end{aligned}$$

can be used to create anisotropic models.

We call a block of the form (36) a *fully coupled multiscale diffusion block*. This block is visualised in Fig. 3. Comparing its form to that of the multichannel diffusion block in Fig. 2, one can see that different architectures use the same activation function design, however, with different motivations.

**Table 1:** The considered diffusion models, along with their variational energies and the resulting network architectures

Model	Variational energy	Diffusion PDE	Explicit scheme/network block	Activation design
Perona–Malik [56], single-channel, isotropic	$E(u) = \int _{\varOmega } \varPsi \left( \text {tr}\left( \varvec{\nabla }u \varvec{\nabla }u^\top \right) \right) \, \mathrm{d}\varvec{x}$	$\partial _t u = \varvec{\nabla }^\top \left( g\left( \left| \varvec{\nabla }u\right| ^2\right) \varvec{\nabla }u\right)$	$\varvec{u}^{k+1} = \varvec{u}^k - \tau \varvec{K}^\top \varvec{\varPhi }\left( \varvec{K} \varvec{u}^k\right)$	Isotropic coupling via structure tensor, scalar multiplication
You and Kaveh [85], single-channel, isotropic	$E(u) = \int _{\varOmega } \varPsi \left( \left( \text {tr}\left( \varvec{H}(u)\right) \right) ^2\right) \, \mathrm{d}\varvec{x}$	$\partial _t u = - \varDelta \left( g\left( \left( \varDelta u\right) ^2\right) \varDelta u\right)$	$\varvec{u}^{k+1} = \varvec{u}^k - \tau \varvec{K}^\top \varvec{\varPhi }\left( \varvec{K} \varvec{u}^k\right)$	Isotropic coupling via Hessian, scalar multiplication
Lysaker et al. [48], single-channel, isotropic	$E(u) = \int _{\varOmega } \varPsi \left( ||\varvec{H}(u)||^2_F\right) \, \mathrm{d}\varvec{x}$	$\partial _t u = - \mathcal {D}^* \left( g\left( ||\varvec{H}(u)||^2_F\right) \mathcal {D} u\right)$ with	$\varvec{u}^{k+1} = \varvec{u}^k - \tau \varvec{K}^\top \varvec{\varPhi }\left( \varvec{K} \varvec{u}^k\right)$	Isotropic coupling via Hessian, scalar multiplication
Gerig et al. [29], coupled multi–channel, isotropic		$\partial _t u_m = \varvec{\nabla }^\top \left( g\left( \sum _{n=1}^M\left| \varvec{\nabla }u_n\right| ^2\right) \varvec{\nabla }u_m\right)$	$\varvec{u}_m^{k+1} = \varvec{u}_m^k - \tau \varvec{K}^\top \varvec{\varPhi }\left( \varvec{u}^k, \varvec{K} \varvec{u}_m^k\right)$	Isotropic coupling via multi-channel structure tensor, scalar multiplication
Weickert and Brox [76], coupled multi–channel, anisotropic		$\partial _t u_m = \varvec{\nabla }^\top \left( g\left( \sum _{n=1}^M\varvec{\nabla }u_n \varvec{\nabla }u_n^\top \right) \varvec{\nabla }u_m\right)$	$\varvec{u}_m^{k+1} = \varvec{u}_m^k - \tau \varvec{K}^\top \varvec{\varPhi }\left( \varvec{u}^k, \varvec{K} \varvec{u}_m^k\right)$	Ansotropic coupling via multi-channel structure tensor, matrix-vector multiplication
Alt and Weickert [3], coupled multiscale, isotropic	$E\left( u\right) = \int _\varOmega \varPsi \left( \text {tr}\int _{0}^{\infty } \left( \mathcal D^{(\sigma )} u\right) \left( {\mathcal {D}}^{(\sigma )} u\right) ^\top \mathrm{d}\sigma \right) \mathrm{d}\varvec{x}$	$\partial _t u - \int _{0}^{\infty }\mathcal {D}^{(\sigma )*} \left( g\left( \int _{0}^{\infty }\left| {\mathcal {D}}^{(\gamma )} u\right| ^2 \mathrm{d}\gamma \right) \mathcal {D}^{(\sigma )} u \right) \mathrm{d}\sigma$	$\varvec{u}^{k+1} = \varvec{u}^k - \tau \sum _{\ell =1}^{L}\omega _\ell \, \varvec{K}_\ell ^\top \varvec{\varPhi } \left( \varvec{u}^k, \varvec{K}_\ell \varvec{u}^k\right)$	Isotropic coupling via multiscale structure tensor, scalar multiplication
Alt and Weickert [3], coupled multiscale, anisotropic	$E\left( u\right) = \int _\varOmega \text {tr}\,\varPsi \left( \int _{0}^{\infty }\left( {\mathcal {D}}^{(\sigma )} u\right) \left( {\mathcal {D}}^{(\sigma )} u\right) ^\top \mathrm{d}\sigma \right) \, \mathrm{d}\varvec{x}$	$\partial _t u = - \int _{0}^{\infty }\mathcal {D}^{(\sigma )*} \left( g\left( \int _{0}^{\infty }\left( \mathcal {D}^{(\gamma )} u\right) \left( {\mathcal {D}}^{(\gamma )} u\right) ^{\top } \mathrm{d}\gamma \right) \mathcal {D}^{(\sigma )} u \right) \mathrm{d}\sigma$	$\varvec{u}^{k+1} = \varvec{u}^k - \tau \sum _{\ell =1}^{L}\omega _\ell \, \varvec{K}_\ell ^\top \varvec{\varPhi } \left( \varvec{u}^k, \varvec{K}_\ell \varvec{u}^k\right)$	Anisotropic coupling via multiscale structure tensor, matrix-vector multiplication

## Discussion

We have seen that shifting the design focus from convolutions to activation functions can yield new insights into CNN design. We summarise all models that we have considered in Table 1 as a convenient overview.

All variational models are rotationally invariant, as they rely on a structural measure which accounts for rotations. This directly transfers to the diffusion model, its explicit scheme, and thus also its network counterpart, resulting in Design Principle 1. Moreover, the different coupling options for models with multiple scales and multiple channels show how a sophisticated activation design can steer the model capacity. This has led to the additional Design Principles 2 and 3 .

The coupling effects are naturally motivated for diffusion, but are hitherto unexplored in the CNN world. While activation functions such as maxout [30] and softmax introduce a coupling of their input arguments, they only serve the purpose of reducing channel information. Even though some works focus on using trainable and more advanced activations [13, 25, 54], the coupling aspect has not been considered so far.

The rotation invariance of the proposed architectures can be approximated efficiently in the discrete setting. For example for second order models, Weickert et al. [77] present $L^2$ stable discretisations with good practical rotation invariance that only require a $3\times 3$ stencil. For models of second order, this is the smallest possible discretisation stencil which still yields consistent results.

In a practical setting with trainable filters, one is not restricted to the differential operators that we have encountered so far. To guarantee that the learned filter corresponds to a rotationally invariant differential operator, one has several options. For example, one can design the filters based on a dictionary of operators which fulfil the rotation invariance property, which are then combined into more complex operators through trainable weights. In a similar manner, one can employ different versions of a base operator which arise from a rotationally invariant operation, e.g. a Gaussian smoothing. We pursue this strategy in our experiments in the following section in analogy to [3].

Apart from the coupling aspect, the underlying network architecture is not modified. This is a stark contrast to the CNN literature where a set of orientations is discretised, requiring much larger stencils for a good approximation of rotation invariance. We neither require involved discretisations, nor a complicated lifting to groups. Thus, we regard the proposed activation function design as a promising alternative to the directional splitting idea.

## Experiments

In the following, we present an experimental evaluation to support our theoretical considerations. To this end, we design trainable multiscale diffusion models for denoising. We compare models with and without coupling activations, and evaluate their performance on differently rotated datasets. This shows that the Design Principle 1 is indeed necessary for rotation invariance.

### Experimental setup

We train the isotropic and anisotropic multiscale diffusion models (32) and (34). Both perform a full coupling of all scales, i.e. they implement Design Principles 1 and 3 . As a counterpart, we train the same multiscale diffusion model with the diffusivity applied to each channel of the discrete derivative operator separately. Thus, the activation is applied independently in each direction. This violates Design Principle 1. Hence, the model should yield worse rotation invariance than the coupled models.

Still, all models implement Design Principle 3 by integrating multiscale information. For an evaluation of the importance of this design principle we refer to [3], where multiscale models outperform their single scale counterparts.

The corresponding explicit scheme for the considered models is given by

41 $$\begin{aligned} \varvec{u}^{k+1} = \varvec{u}^k - \tau \sum _{\ell =1}^{L}\omega _\ell \, \varvec{K}_\ell ^\top \varvec{\varPhi } \left( \varvec{u}^k, \varvec{K}_\ell \varvec{u}^k\right) \end{aligned}$$

The choice for $\omega _\ell$ is set to $\sigma _{\ell +1}-\sigma _{\ell }$.

As differential operators $\varvec{K}_\ell$, we choose weighted, Gaussian smoothed gradients $\beta _\ell \varvec{\nabla }_{\sigma _\ell }$ on each scale $\sigma _\ell$. The application of a smoothed gradient to an image via $\varvec{\nabla }_{\sigma } u = G_{\sigma } *\varvec{\nabla }u$ is equivalent to computing a Gaussian convolution with standard deviation $\sigma$ of the image gradient. Moreover, we weight the differential operators on each scale by a scale-adaptive, trainable parameter $\beta _\ell$.

A discrete set of $L=8$ scales is determined by an exponential sampling between a minimum scale of $\sigma _{\text {min}}=0.1$ and a maximum one of $\sigma _{\text {max}}=10$. This yields discrete scales [0.1, 0.18, 0.32, 0.56, 1.0, 1.77, 3.16, 5.62].

To perform edge-preserving denoising, we choose the exponential Perona–Malik [56] diffusivity

42 $$\begin{aligned} g(s^2) = \exp \left( -\frac{s^2}{2\lambda ^2}\right) . \end{aligned}$$

It attenuates the diffusion at locations where the argument exceeds a contrast parameter $\lambda$. This parameter is trained in addition to the scale-adaptive weights.

Moreover, we train the time step size $\tau$ and we use 10 explicit steps with shared parameter sets. This amounts to a total number of 10 trainable parameters: $\tau$, $\lambda$, and $\beta _1$ to $\beta _8$.

In the practical setting, a discretisation with good rotation invariance is crucial. We use the nonstandard finite difference discretisation of Weickert et al. [77]. It implements the discrete divergence term

43 $$\begin{aligned} \varvec{A}(\varvec{u}^k) = \varvec{K}_\ell ^\top \varvec{\varPhi } \left( \varvec{u}^k, \varvec{K}_\ell \varvec{u}^k\right) \end{aligned}$$

on a stencil of size $3\times 3$. For isotropic models, it has a free parameter $\alpha \in \left[ 0, \frac{1}{2}\right]$ which can be tuned for rotation invariance, with an additional parameter $\gamma \in \left[ 0, 1\right]$ for anisotropic ones. We found that in the denoising case, the particular choice of these two parameters constitutes a trade-off between performance and rotation invariance.

We train all models on a synthetic dataset which consists of greyscale images of size $256\times 256$ with values in the range $\left[ 0, 255\right]$. Each image contains 20 randomly placed white rectangles of size $140\times 70$ on a black background. The rectangles are all oriented along a common direction, which creates a directional bias within the dataset. The training set contains 100 images and is oriented with an angle of $30^\circ$ from the *x*-axis. As test datasets, we consider rotated versions of a similar set of 50 images. The rotation angles are sampled between $0^\circ$ and $90^\circ$ in steps of $5^\circ$. To avoid an influence of the image sampling on the evaluation, we exclude the axis-aligned datasets.

To train the models for the denoising task, we add noise of standard deviation 60 to the clean training images and minimise the Euclidean distance to the ground truth images. We measure the denoising quality in terms of peak-signal-to-noise ratio (PSNR). All models are trained for 250 epochs with the Adam optimiser [37] with standard settings and a learning rate of 0.001. One training epoch requires 50 seconds on an *NVIDIA GeForce GTX 1060 6GB*, and the evaluation on one of the test sets requires 7 seconds.

**Fig. 4:**
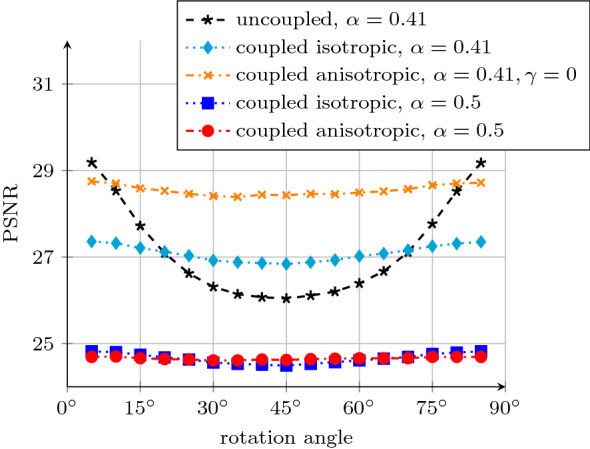
Denoising quality on differently rotated versions of the test dataset. The models have been trained on a dataset with $30^\circ$ orientation. The models with coupling approximate rotation invariance significantly better than the uncoupled model

**Fig. 5:**
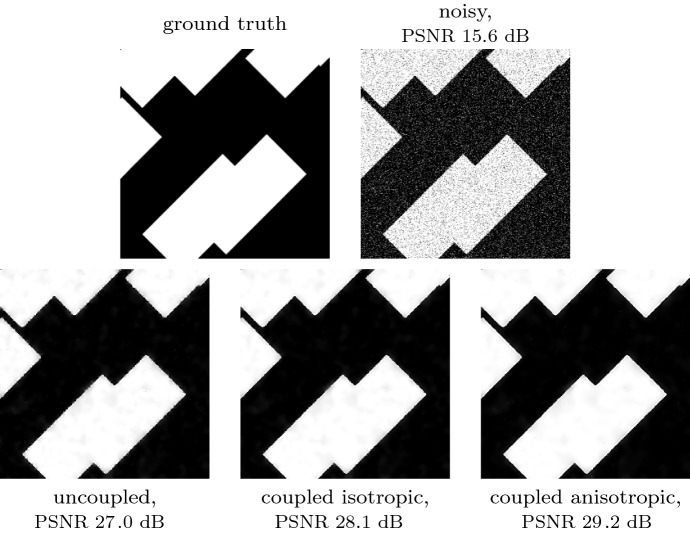
Visual comparison of denoising results for a rotation angle of $45^\circ$. The coupled models achieve better denoising quality as they generalise better to the rotated data

### Evaluation

A rotationally invariant model should produce the same PSNR on all rotations of the test dataset. Thus, in Fig. 4 we plot the PSNR on the test datasets against their respective rotation angle.

We see that the fluctuations within both anisotropic and isotropic coupled models are much smaller than those within the uncoupled model. A choice of $\alpha =0.41$ and $\gamma =0$ yields a good balance between performance and rotation invariance. However, rotation invariance can also be driven to the extreme: A choice of $\alpha =0.5$, which renders the choice of $\gamma$ irrelevant, eliminates rotational fluctuations almost completely, but also drastically reduces the quality. The reason for this is given by Weickert et al. [77]: A value of $\alpha =0.5$ decouples the image grid into two decoupled checkerboard grids which do not communicate except at the boundaries.

For the balanced choice of $\alpha =0.41$, the anisotropic model consistently outperforms the isotropic one, as it can smooth along oriented structures. As the uncoupled model can only do this for structures which are aligned with the *x*- and *y*-axes, it performs better the closer the rotation is to $0^\circ$ and $90^\circ$, respectively. Hence, it performs worst for a rotation angle of $45^\circ$. Thus, it does not achieve rotation invariance.

We measure the rotation invariance in terms of the variance of the test errors over the rotation angles. While the isotropic and anisotropic coupled models with $\alpha =0.41$ achieve variances of 0.035 dB and 0.014 dB, the uncoupled model suffers from a variance of 1.25 dB. The extreme choice of $\alpha =0.5$ even reduces the variances of the coupled models to 0.013 dB and $8.7\cdot 10^{-4}$ dB, respectively.

A visual inspection of the results in Fig. 5 supports this trend. Therein, we present the denoised results on an example from the test data set with $45^\circ$ orientation. The uncoupled model suffers from ragged edges as the training on the differently rotated dataset has introduced a directional bias. The coupled isotropic model preserves the edges far better, and the coupled anisotropic model can even smooth along them to obtain the best reconstruction quality.

These findings show that the coupling effect leads to significantly better rotation invariance properties.

## Conclusions

We have seen that the connection between diffusion and neural networks allows to bring novel concepts for rotation invariance to the world of CNNs. The models which we considered inspire different activation function designs, which we summarise in Table 1.

The central design principle for rotation invariance is a coupling of operator channels. Diffusion models and their associated variational energies apply their respective nonlinear design functions to rotationally invariant quantities based on a coupling of multi-channel differential operators. Thus, the activation function as their neural counterpart should employ this coupling, too. Moreover, coupling image channels or scales in addition allows to create anisotropic models with better measures for structural information.

This strategy provides an elegant and minimally invasive modification of standard architectures. Thus, coupling activation functions constitute a promising alternative to the popular network designs of splitting orientations and group methods in orientation space. Evaluating these concepts in practice and transferring them to more general neural network models are part of our ongoing work.
